# Association of red cell distribution width/albumin ratio with intraoperative blood transfusion in cervical cancer patients

**DOI:** 10.1371/journal.pone.0277481

**Published:** 2022-11-18

**Authors:** Ji-Hoon Sim, Dong-Min Jang, Hyun-Seok Cho, Jong Yeon Park, Woo-Jong Choi

**Affiliations:** Department of Anesthesiology and Pain Medicine, Asan Medical Center, University of Ulsan College of Medicine, Seoul, Republic of Korea; University of Cape Town Faculty of Science, SOUTH AFRICA

## Abstract

**Background:**

Although minimally invasive surgical techniques have reduced intraoperative bleeding, the risk of transfusion exists. However, few studies have evaluated risk factors for transfusion in radical hysterectomy. We aimed to evaluate the association between preoperative red cell distribution width/albumin ratio (RDW/albumin) and transfusion in cervical cancer patients.

**Methods:**

We analyzed 907 patients who underwent radical hysterectomy between June 2006 and February 2015. Logistic regression and Cox regression analyses were performed to determine the risk factors for transfusion and mortality at 5-year and overall. Net reclassification improvement (NRI) and integrated identification improvement (IDI) analyses were performed to verify the improvement of the intraoperative transfusion model upon the addition of RDW/albumin.

**Results:**

RDW/albumin was an independent risk factor for transfusion (odds ratio [OR]: 1.34, 95% confidence interval [CI]: 1.02–1.77, p = 0.035). Additionally, body mass index, operation time, laparoscopic surgery, total fluids, and synthetic colloid were risk factors for transfusion. RDW/albumin was an independent risk factor for 5-year mortality (hazard ratio [HR]: 1.51, 95% CI: 1.07–2.14, p = 0.020), and overall mortality (HR: 1.48, 95% CI: 1.06–2.07, p = 0.021). NRI and IDI analyses showed the discriminatory power of RDW/albumin for transfusion (p<0.001 and p = 0.046, respectively).

**Conclusions:**

RDW/albumin might be a significant factor in transfusion and mortality in cervical cancer patients.

## Introduction

Cervical cancer is one of the most common types of gynecological cancers, was the fourth most common neoplasm among females worldwide in 2018, and is the leading cause of cancer-related death [1]. The incidence of cervical cancer is 13.1 per 100,000 individuals, and the main treatment is radical hysterectomy, which is one of the major abdominal surgeries [1–3]. Recent advances in minimally invasive techniques such as laparoscopy and robotic surgery have reduced intraoperative bleeding [4, 5]. However, the risk factors for intraoperative transfusion remain to be clarified.

The red cell distribution width (RDW) is a biological marker that describes the variability in red blood cell (RBC) size [6]. RDW has been reported as a simple and objective indicator of patient survival and complications in various diseases [7]. RDW has been reported to be associated with iron deficiency anemia as well as liver disease and various inflammatory diseases, which may be associated with intraoperative blood loss and transfusions and poor surgical outcomes [8, 9]. The serum albumin level also has a clear prognostic value in predicting postoperative surgical outcomes [10]. Hypoalbuminemia significantly influences the length of hospital stay and complication rates, specifically surgical site infection [11, 12], and has been reported to be associated with intraoperative blood transfusion [13]. Recently, it has been reported that the ratio of RDW/albumin ratio, which is the combined index of RDW and albumin, is associated with 60-day mortality in patients with acute respiratory distress syndrome [14]. However, its clinical usefulness as a risk factor for intraoperative transfusion and surgical outcomes has not yet been evaluated. Therefore, we evaluated the association between preoperative RDW/albumin and intraoperative transfusion and surgical prognosis in patients who underwent radical hysterectomy for cervical cancer.

## Materials and methods

### Study design and patients

Patients diagnosed with cervical cancer according to the 10th Amendment to the International Classification of Diseases guidelines who underwent open or laparoscopic radical hysterectomy between June 2006 and February 2015 were enrolled in this study. The institutional review board of Asan Medical Center (Republic of Korea; protocol number: 2020–1779) approved this retrospective study and waived the need for written informed consent. This study conformed with The Code of Ethics of the World Medical Association (Declaration of Helsinki). The study included adult patients over 18 years of age who were diagnosed with cervical cancer. The exclusion criteria were as follows: age < 18 or ≥ 80 years; hematologic diseases and other malignancies; ongoing use of anticoagulants, such as warfarin and antiplatelet agents; and incomplete data or missing RDW or serum albumin values.

### Anesthetic technique

For general anesthesia, we used an intravenous bolus injection of thiopental sodium (4–5 mg/kg) or propofol (1.5–2 mg/kg). Before tracheal intubation, 0.6–1.0 mg/kg of rocuronium was injected intravenously as a bolus, and maintenance of anesthesia was performed with sevoflurane 1.5–3 vol% in 50% nitrous oxide/oxygen. After insertion of the arterial lines, invasive arterial blood pressure monitoring was routinely performed. The patients were mechanically ventilated with a tidal volume of 6–8 mL/kg, and the end-expiratory carbon dioxide partial pressure was adjusted to maintain a value of 35–40 mmHg. During anesthesia, crystalloid solutions (plasma solution or Ringer’s lactate solution) or colloid solutions (5% albumin or synthetic colloids [Voluven^®^; Fresenius Kabi, Bad Homburg, Germany]) were administered. The total volume of synthetic colloid administered did not exceed 20 mL/kg. Preoperative iron or B12 supplementation and intraoperative use of antifibrinolytic agents were not routinely used. During surgery, when the plasma hemoglobin (Hb) level was less than 8 g/dL, packed RBC transfusion was performed, and the Hb level was maintained at >10 g/dL in patients with ischemic heart disease. Vasopressors such as ephedrine or phenylephrine were administered when the mean arterial blood pressure was less than 65 mmHg, and inotropic agents such as norepinephrine were administered when the vasopressor was ineffective according to the clinical judgment of an anesthesiologist.

### Clinical data collection and outcome assessments

Demographic data and pre-, intra-, and postoperative variables were collected from an electronic medical record system. Demographic and preoperative data included age, weight, height, body mass index (BMI), and American Society of Anesthesiologists (ASA) status. The cancer staging was based on the International Federation of Gynecology and Obstetrics (FIGO) stage classification. Data on comorbid diseases such as diabetes mellitus, hypertension, liver disease, and kidney disease were also collected.

Laboratory values included preoperative white blood cell count, Hb, platelet count, glucose, albumin, creatinine, and RDW/albumin. RDW was measured by sheath-flow DC method using XN-series (Sysmex, Japan) equipment. Albumin was measured by Colorimetric assay-bromcresol purple method using Cobas 8000 (F. Hoffman-La Roche Ltd, Austria) analytics. RDW/albumin was calculated using the following formula: [RDW (%)] / [albumin (g/dL)]. The total blood counts of all patients were determined preoperatively, < 2 days after admission, and prior to treatment.

Intraoperative variables included operation time, laparoscopic surgery, total fluids, synthetic colloid use, and RBC transfusion. Postoperative variables included histology, chemotherapy, and radiation therapy. The histopathological records of the patients were examined and classified into four categories: 1 = squamous cell carcinoma, 2 = adenocarcinoma, 3 = adenosquamous carcinoma, 4 = small-cell and neuroendocrine carcinoma. Postoperative hospital days, intensive care unit (ICU) admission, 5-year mortality (calculated from the date of surgery to 5-year follow-up), and overall mortality (determined from the date of surgery to the last follow-up) records were also collected.

The primary aims were to analyze the risk factors for intraoperative transfusion and to evaluate the association between preoperative RDW/albumin and intraoperative transfusion. The secondary aim was to determine whether RDW/albumin was an independent risk factor for surgical outcomes such as hospital stay, ICU admission, 5-year, and overall mortality.

### Statistical analysis

Categorical data were analyzed using the chi-square test or Fisher’s exact test, and continuous data were evaluated using an independent t-test or Mann–Whitney U test. Data are appropriately presented as mean and standard deviation, median of the quartile range, or numbers with proportions. We used multivariable logistic regression analysis to determine the risk factors for intraoperative transfusion. All variables with p-values less than 0.1 in the univariate analysis were included in the multivariate analysis. Cox regression analysis was also used to evaluate the adjusted risk ratio of the 5-year mortality risk factor. In addition, the predictive value of preoperative RDW/albumin for discriminating intraoperative transfusion was evaluated through receiver operating characteristic (ROC) curve and analysis of net reclassification improvement (NRI) and integrated discrimination improvement (IDI). All p-values < 0.05 were considered statistically significant. Data manipulation and statistical analyses were performed using IBM SPSS Statistics for Windows, version 22.0 (IBM Corporation, Armonk, NY, USA).

## Results

**Fig 1:**
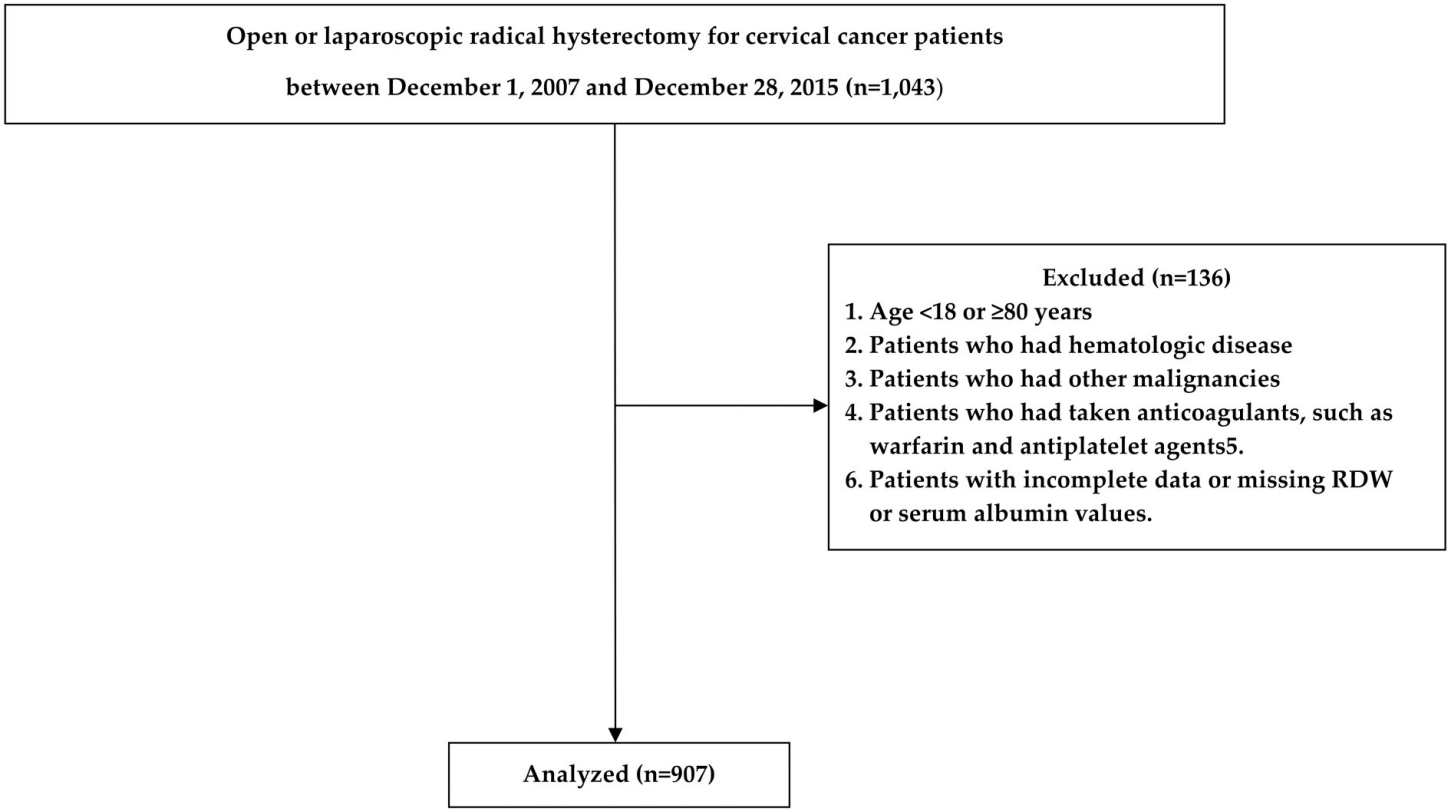
Flowchart of the retrospective study.

Of the 1,043 enrolled patients, 136 were excluded because they did not fulfill the study criteria. Hence, a total of 907 patients were enrolled in this study (Fig 1).

Table 1 shows the demographic data, perioperative variables, and surgical outcomes of the study population. Most of the patients who participated in this study were classified as ASA 1 (25.0%) and ASA 2 (73.5%) and FIGO stages 1A (12.0%), 1B (68.7%), 2A (10.4%), and 2B (8.9%). Histologically, most were squamous cell carcinomas (69.3%) and adenocarcinomas (23.9%), whereas small-cell and neuroendocrine carcinomas were 19 (2.1%).

**Table 1:** Demographic data and perioperative variables of the study population.

	Study population (N = 907)
**Perioperative variables**	
Age (years)	47.70 ± 11.49
BMI (kg.m^-2^)	23.44 ± 3.28
DM	43 (4.7)
HTN	122 (13.5)
Liver disease	5 (0.6)
Kidney disease	3 (0.3)
Other chronic disease	18 (2.0)
ASA status	
ASA 1	227 (25.0)
ASA 2	667 (73.5)
ASA 3	13 (1.5)
FIGO stage	
Stage 1A	109 (12.0)
Stage 1B	623 (68.7)
Stage 2A	94 (10.4)
Stage 2B	81 (8.9)
**Laboratory variables**	
White blood cell, 10^3^/uL	6.44 ± 2.08
Hemoglobin, g/dL	12.32 ± 1.38
Platelets, 10^9^/L	257.46 ± 63.08
Glucose, mg/ dL	111.90 ± 32.87
Albumin, g/dL	3.98 ± 0.36
Creatinine, mg/dL	0.66 ± 0.23
RDW/albumin	3.40 ± 0.66
**Intraoperative variables**	
Operation time, min	287.94 ± 62.51
Total fluids, mL/kg	61.67 ± 24.83
Synthetic colloid use	589 (64.9)
Laparoscopic surgery	711 (78.4)
**Postoperative variables**	
Histology	
Squamous cell carcinoma	629 (69.3)
Adenocarcinoma	217 (23.9)
Adenosquamous carcinoma	42 (4.6)
Small-cell and neuroendocrine carcinoma	19 (2.1)
Postoperative CTx	391 (43.1)
Postoperative RTx	407 (44.9)
**Transfusion**	
Preoperative RBC transfusion	26 (2.9)
Intraoperative RBC transfusion	307 (33.8)
Intraoperative RBC unit	0.79 ± 1.47
Postoperative RBC transfusion	89 (9.8)
**Surgical outcomes**	
Hospital stay	10.30 ± 4.61
Hospital stay (≥ 14 days)	57 (6.3)
ICU admission	11 (1.2)
5-year mortality	33 (3.6)
Overall mortality	42 (4.6)

BMI: body mass index; DM: diabetes mellitus; HTN: hypertension; ASA: American Society of Anesthesiologists; FIGO: International Federation of Gynecology and Obstetrics; RDW: red cell distribution width; RBC: red blood cells; CTx: chemotherapy; RTx: radiation therapy; ICU: intensive care unit.

Values are expressed as mean ± standard deviation or n (proportion).

The incidence of intraoperative transfusion was 33.8% (307/907) and the average packed RBC transfusion volume was 0.79 units. The average length of hospital stay was 10.30 days, ICU admission rate was 1.2% (12/907), 5-year mortality was 3.6% (33/907), and overall mortality was 4.6% (42/907; Table 1).

### Primary aims

In the multivariate analysis, preoperative RDW/albumin was an independent risk factor for intraoperative transfusion (odds ratio [OR]: 1.34, 95% confidence interval [CI]: 1.02–1.77, p = 0.035; Table 2). Additionally, BMI (OR: 1.08, 95% CI: 1.01–1.15, p = 0.020), operation time (OR: 1.00, 95% CI: 1.00–1.01, p = 0.015), laparoscopic surgery (OR: 0.30, 95% CI: 0.20–0.45, p<0.001), total fluids (OR: 1.05, 95% CI: 1.04–1.06, p<0.001), and synthetic colloid use (OR: 3.50, 95% CI: 2.18–5.62, p<0.001) were risk factors for intraoperative transfusion (Table 2).

**Table 2:** Univariate and multivariate logistic regression analysis of intraoperative transfusion.

	Univariate			Multivariate		
	OR	95% CI	P	OR	95% CI	P
RDW/albumin	1.53	1.25–1.89	< 0.001	1.34	1.02–1.77	0.035
Hemoglobin						
<13	1.00 (Ref.)			1.00 (Ref.)		
≥13	0.06	0.01–0.50		0.13	0.01–1.11	
Age (years)	1.00	0.99–1.01	0.745			
BMI (kg.m^-2^)	0.97	0.93–1.01	0.107	1.08	1.01–1.15	0.020
DM	0.75	0.38–1.48	0.400			
HTN	0.95	0.63–1.42	0.790			
ASA status			0.813			
ASA 1,2	1.00 (Ref.)					
ASA 3	0.87	0.26–2.84				
FIGO stage			< 0.001			0.237
Stage 1A	1.00 (Ref.)			1.00 (Ref.)		
Stage 1B,2A,2B	2.67	1.60–4.48		1.45	0.78–2.67	
Operation time (min)	1.02	1.01–1.02	< 0.001	1.00	1.00–1.01	0.015
Laparoscopic surgery	0.29	0.21–0.40	< 0.001	0.30	0.20–0.45	< 0.001
Total fluids (mL/kg)	1.06	1.05–1.07	< 0.001	1.05	1.04–1.06	< 0.001
Synthetic colloid use	8.52	5.66–12.83	< 0.001	3.50	2.18–5.62	< 0.001
Histology			0.399			
Squamous cell carcinoma	1.00 (Ref.)					
Adenocarcinoma	0.86	0.62–1.20	0.372			
Adenosquamous carcinoma	0.58	0.28–1.20	0.139			
Small-cell carcinoma and neuroendocrine carcinoma	1.08	0.42–2.77	0.878			

OR: odds ratio; CI: confidence interval; RDW: red cell distribution width; BMI: body mass index; DM: diabetes mellitus; HTN: hypertension; ASA: American Society of Anesthesiologists; FIGO: International Federation of Gynecology and Obstetrics.

Values are expressed as mean ± standard deviation, median (interquartile range), or n (proportion).

The addition of RDW/albumin to the clinical model for intraoperative transfusion, consisting of preoperative hemoglobin, BMI, operation time, total fluids, synthetic colloid use, and laparoscopic surgery, showed no significant improvement in the area under the curve (p = 0.190) but significant discriminative power in NRI analysis (0.268, 95% CI: 0.138–0.399, p<0.001) and IDI analysis (0.006, 95% CI: 0.000–0.012, p = 0.046; Table 3).

**Table 3:** Improvement in AUC and NRI by addition of RDW/albumin to clinical predictive models.

		AUC (95% CI)	P-value	NRI (95% CI)	P-value	IDI (95% CI)	P-value
Transfusion	Model 1*	0.866 (0.841–0.890)					
	Model 1* + RDW/albumin	0.869 (0.845–0.893)	0.190	0.268 (0.138–0.399)	<0.001	0.006 (0.000–0.012)	0.046

*Model 1 = hemoglobin + BMI + operation time + total fluids + synthetic colloid use + laparoscopic surgery

AUC: area under the curve; CI: confidence interval; NRI: net reclassification improvement; IDI: integrated discrimination improvement; RDW: red cell distribution width; BMI: body mass index;

Values are expressed as mean ± standard deviation, median (interquartile range), or n (proportion).

### Secondary aims

In the Cox regression analysis, preoperative RDW/albumin was an independent risk factor for 5-year mortality (hazard ratio [HR]: 1.50, 95% CI: 1.04–2.17, p = 0.033; Table 4). Moreover, histology of small-cell and neuroendocrine carcinoma was a risk factor for 5-year mortality (HR: 10.09, 95% CI: 3.69–27.59, p<0.001; Table 4). Preoperative RDW/albumin was also risk factor for a hospital stay ≥ 14 days (OR: 1.39, 95% CI: 1.06–1.83, p = 0.018) and overall mortality (HR: 1.48, 95% CI: 1.06–2.07, p = 0.021) (Table 5).

**Table 4:** Cox regression analysis of 5-year mortality.

	Univariate			Multivariate		
	HR	95% CI	P	HR	95% CI	P
RDW/albumin	1.49	1.04–2.13	0.030	1.50	1.04–2.17	0.033
Hemoglobin						
<13	1.00 (Ref.)			1.00 (Ref.)		
≥13	0.06	0.01–0.50		0.13	0.01–1.11	
Age (years)	0.98	0.95–1.01	0.244			
BMI (kg.m^-2^)	0.88	0.79–0.99	0.039	0.90	0.80–1.01	0.069
DM	0.62	0.09–4.50	0.639			
HTN	1.15	0.45–2.97	0.771			
ASA status			0.434			
ASA 1,2	1.00 (Ref.)					
ASA 3	2.22	0.31–16.07				
FIGO stage			0.143			0.179
Stage 1A	1.00 (Ref.)			1.00 (Ref.)		
Stage 1B,2A,2B	4.42	0.61–32.02		3.94	0.54–28.87	
Total fluids (mL/kg)	1.01	1.00–1.02	0.130			
Synthetic colloid use	1.08	0.53–2.22	0.834			
Histology			0.003			< 0.001
Squamous cell carcinoma	1.00 (Ref.)			1.00 (Ref.)		
Adenocarcinoma	1.56	0.70–3.49	0.279	1.59	0.71–3.57	0.262
Adenosquamous carcinoma	1.77	0.41–7.61	0.444	1.77	0.41–7.60	0.446
Small-cell carcinoma and neuroendocrine carcinoma	11.17	4.14–30.13	< 0.001	10.09	3.69–27.59	< 0.001
Preoperative transfusion	1.08	0.15–7.94	0.937			
Intraoperative transfusion	1.29	0.64–2.58	0.481			
Postoperative transfusion	0.97	0.30–3.18	0.970			

HR: hazards ratio; CI: confidence interval; RDW: red cell distribution width; BMI: body mass index; DM: diabetes mellitus; HTN: hypertension; ASA: American Society of Anesthesiologists; FIGO: International Federation of Gynecology and Obstetrics.

Values are expressed as mean ± standard deviation, median (interquartile range), or n (proportion).

**Table 5:** Transfusion and surgical outcomes adjusted by RDW/albumin.

	Univariate		Multivariate	
	OR (95% CI)	P-value	OR (95% CI)*	P-value
RBC transfusion	1.53 (1.25–1.89)	< 0.001	1.34 (1.02–1.77)	0.035
Hospital stay (≥14 days)	1.52 (1.20–1.94)	< 0.001	1.39 (1.06–1.83)	0.018
ICU admission	1.56 (0.85–2.87)	0.149	1.42 (0.71–2.82)	0.322
	HR (95% CI)	P-value	HR (95% CI)**	P-value
5-year mortality	1.49 (1.04–2.13)	0.030	1.50 (1.04–2.17)	0.033
Overall mortality	1.45 (1.04–2.01)	0.028	1.48 (1.06–2.07)	0.021

*Adjusted for hemoglobin, BMI, FIGO stage, operation time, total fluids, synthetic colloid use, and laparoscopic surgery

**Adjusted for BMI, FIGO stage, and histology

OR: odds ratio; HR: hazards ratio; CI: confidence interval; RDW: red cell distribution width; RBC: red blood cells; ICU: intensive care unit; BMI: body mass index; ASA: American Society of Anesthesiologists; FIGO: International Federation of Gynecology and Obstetrics

Values are expressed as mean ± standard deviation, median (interquartile range), or n (proportion).

## Discussion

Our study demonstrated that preoperative RDW/albumin was an independent risk factor for intraoperative transfusion in patients who underwent radical hysterectomy for cervical cancer. Preoperative RDW/albumin was also a risk factor for prolonged hospital stay, higher 5-year and overall mortality. In addition, RDW/albumin showed discriminative power for transfusion. This suggests that preoperative RDW/albumin might be a strong risk factor for transfusion and surgical outcomes in cervical cancer patients.

Radical hysterectomy for cervical cancer is associated with significant bleeding and intraoperative transfusion [15]. Benjamin et al. reported that blood transfusions were performed in 44%–91% of patients who underwent open radical hysterectomy [16]. More recent studies have reported an estimated blood loss of 500–800 mL for open radical hysterectomy [17] and 100–300 mL for laparoscopic or robotic radical hysterectomy [18, 19]. Although minimally invasive surgical techniques are associated with reduced bleeding and transfusion requirements [4, 5], these state-of-the-art technologies may not be available in underdeveloped countries, where cervical cancer is most prevalent and vulnerable. In addition, a recent large-scale prospective study reported that minimally invasive surgery had poorer disease-free survival and overall survival than open surgery [20]. Therefore, intraoperative bleeding and transfusions, which are primarily associated with extensive and aggressive surgical resection, are still issues that need to be dealt with. However, there are few studies on the risk factors for intraoperative bleeding and transfusions in cervical cancer surgery. A 2019 study reported that clinical stage, age, BMI, and laparoscopic surgery predict intraoperative bleeding and transfusion during early cervical cancer surgery [21]. Our study is clinically meaningful as the first major investigation to evaluate the association of a novel biomarker, RDW/albumin, with intraoperative transfusion and surgical prognosis.

In the multivariable logistic regression analysis, RDW/albumin, BMI, operation time, laparoscopic surgery, total fluids, and synthetic colloid use were also risk factors for intraoperative transfusion. Among the risk factors for transfusion, BMI and obesity remain controversial. Elke and colleagues have reported that obesity is associated with an increased risk of blood transfusion [22]. However, Nam and colleagues found that obesity was protective for transfusion [23] They reported that hemoglobin could be significantly affected by relatively small blood loss in low-weight patients who have less blood volume than overweight patients. Operation time reflects the surgical complexity and severity of disease with probably higher intraoperative blood loss, and therefore might be related to higher levels of blood transfusion and a longer hospital stay [24]. Previous studies have reported that laparoscopic surgery is associated with less blood loss [3, 4, 21], which was consistent with our results. Synthetic colloid administration has been reported to be associated with coagulopathy and blood transfusion [25]. However, since the amount of synthetic colloid administered was limited to 20 mL/kg, the relationship between fluid management and blood transfusion is thought to be a consequence of the increased crystalloid and colloid use due to bleeding.

In the current study, the association between preoperative RDW/albumin and transfusion seems to be linked to the properties of RDW and albumin that reflect the patient’s inflammatory response and nutritional status. Inflammatory reaction and release of cytokines can increase the intraoperative bleeding risk by creating an abnormal clotting system and hypercoagulable condition [26]. Nutritional deficiencies and inflammatory status may exacerbate the disease severity [27], making surgery difficult and increasing the risk of bleeding [28]. Recent studies have shown that elevated RDW may be associated with a risk of bleeding [8, 9]. The mechanism by which RDW increases is not yet clear, however, it is predicted to be triggered by anemia, inflammation, and oxidative stress [29]. Increased RDW is a sign of a nutritional deficit, such as a deficiency of iron, folic acid, or vitamin B-12, which can indicate macrocytic anemia and may increase intraoperative transfusion [30]. Inhibition of erythrocyte maturation by inflammatory cytokines can also lead to the requirement of transfusions by causing abnormal erythropoietin function and coagulation system [31]. Elevated RDW levels have been reported to be associated with peripheral vascular disease, which may be associated with increased procedural complications and bleeding. [32] Hypoalbuminemia may induce a hypovolemic state due to low oncotic pressure [33]. In patients with hypoalbuminemia and consequent hypovolemia, the effective circulating volume may be further reduced if intraoperative blood loss occurs [34, 35]. Therefore, patients with hypoalbuminemia with reduced effective circulating volume are more likely to need a packed RBC transfusion [13].

Our study demonstrated that preoperative RDW/albumin was a risk factor for surgical outcomes such as prolonged hospital stay, 5-year, and overall mortality, which is in line with a previous study on patients with acute respiratory distress syndrome [14]. It is also consistent with studies that show that RDW may be associated with surgical prognosis in numerous cancer patients [7]. Hypoalbuminemia has also been reported to be associated with surgical outcomes [36]. A recent randomized clinical trial demonstrated that long-term albumin administration in patients with decompensated cirrhosis improved overall survival and complications [37].

There are some limitations to our study. First, our study is retrospective in nature; thus, the possibility of undocumented factors being reported, potential bias associated with patient selection, and recall bias existed. However, we tried to reduce the impact of confounding factors by adjusting for variables that could affect the outcome. Second, our data consisted mostly of a single ethnic group within Korea, therefore, the results may have been biased due to homogeneous groups. Therefore, our results may differ from those of studies conducted by other institutions or countries, and further research involving different ethnic groups is needed. Third, to date, no study has reported the cutoff value of RDW/albumin for intraoperative transfusion and mortality. More well-designed studies on various diseases are required for accurate validation of preoperative RDW/albumin cutoff value that could predict surgical outcomes. Fourth, several diseases, including liver and renal dysfunction, can result in decreased albumin levels and increased RDW, which can be a significant limitation of the study. However, in our study, the age of the patient group was a relatively young 40-year-old woman with few comorbidities and only 8 patients with liver and kidney disease. Therefore, it is judged that the above-mentioned diseases have little effect on the results of our study.

In conclusion, preoperative RDW/albumin might be a significant risk factor for intraoperative transfusion and mortality in patients who underwent radical hysterectomy for cervical cancer. These results suggest that preoperative RDW/albumin provides clinically useful information on intraoperative transfusion and surgical prognosis in cervical cancer patients.
